# Chinese Preschool Children’s Socioemotional Development: The Effects of Maternal and Paternal Psychological Control

**DOI:** 10.3389/fpsyg.2017.01818

**Published:** 2017-10-18

**Authors:** Shufen Xing, Xin Gao, Xinxin Song, Marc Archer, Demao Zhao, Mengting Zhang, Bilei Ding, Xia Liu

**Affiliations:** ^1^Department of Psychology, Capital Normal University, Beijing, China; ^2^Qingdao Mental Health Center, Qingdao, China; ^3^School of Public Health, Zhejiang University, Hangzhou, China; ^4^Institute of Developmental Psychology, Beijing Normal University, Beijing, China

**Keywords:** psychological control, socioemotional development, behavioral problems, prosocial behaviors, preschool children

## Abstract

The present study examined the relative prediction and joint effects of maternal and paternal psychological control on children’s socioemotional development. A total of 325 preschool children between the ages of 34 and 57 months (*M* = 4 years 2 months) and their parents participated in the study. Fathers and mothers, respectively, reported their levels of psychological control and mothers evaluated the socioemotional development of children using two indicators (i.e., behavioral problems and prosocial behaviors). The results indicated that the relative predictive effects of maternal and paternal psychological control on children’s socioemotional development differed. Specifically, maternal psychological control was a significant predictor of children’s behavioral problems and prosocial behaviors, whereas the levels of paternal psychological control were unrelated to children’s socioemotional development. With regard to the combined effects of maternal and paternal psychological control, the results of ANOVAs and simple slope analysis both indicated that children would be at risk of behavioral problems as long as they had one highly psychologically controlling parent. High levels of paternal psychological control were associated with increased behavioral problems of children only when maternal psychological control was low. However, the association between maternal psychological control and children’s behavioral behaviors was significant, despite paternal psychological control.

## Introduction

Parental psychological control refers to a set of intrusive parenting behaviors characterized by manipulation of children’s inner world such as guilt induction, love withdrawal, shaming, constraining children’s expressions, and stifling autonomy (Schaefer, 1965; Barber, 1996; Gao et al., 2016). Children with psychologically controlling parents feel forced to act, think, or feel in ways dictated by their parents (Soenens and Vansteenkiste, 2010). The developmental niche model holds that parenting behaviors are the pivotal subsystem of children’s physical and mental development (Super and Harkness, 1986). Psychological control serves as an important indicator of parenting quality; high levels of parental psychological control are associated with children’s social and psychological dysfunction (Barber and Harmon, 2002). For example, studies have demonstrated that children with highly psychologically controlling parents report less prosocial behaviors (Clark et al., 2015), more relational aggressive behaviors (Kuppens et al., 2013), as well as higher levels of anxiety (Settipani et al., 2013), and depression (El-Sheikh et al., 2010). Therefore, high parental psychological control represents an important risk factor in the socioemotional development of children.

### The Relative Prediction of Maternal and Paternal Psychological Control

A number of existing research about parental psychological control has focused on mothers (e.g., Mills and Rubin, 1998; El-Sheikh et al., 2010) or combined maternal and paternal psychological control into an aggregated construct of parenting behaviors (e.g., Wang et al., 2007; Lai et al., 2014). The few studies that have examined the separate effects of maternal and paternal psychological control on children’s socioemotional development have yielded inconsistent results. For example, Hart et al. (2000) found that among Russian preschool children only maternal – and not paternal – psychological control had a significant association with children’s aggressive behaviors. Laible and Carlo (2004) indicated that low levels of rigid control from mothers were related to children’s sympathy, social competence, and self-worth, whereas paternal control was not a predictor of children’s adjustment. Those results were consistent with the understanding that maternal parenting behaviors play the dominant and primary role in children’s development. However, a recent longitudinal study has determined that both maternal and paternal psychological controls significantly predict changes in children’s behavioral problems (Lansford et al., 2014). The relevant findings support quite different conclusions, suggesting a need for further examination of this matter.

Another question that requires further exploration is whether the adverse effects of parental psychological control may be generalized to different cultures. Some studies have suggested that parental psychological control may be more common in Eastern countries, such as China, than in Western societies (Soenens and Vansteenkiste, 2010). In addition, psychological control could have distinct effects in different cultures. For example, shaming, which generally seems negative in West, is used as a moral socialization mechanism to teach children the social norms and requirements in Chinese culture (Helwig et al., 2014). The specific effects of parental psychological control in Chinese culture merit attention.

Finally, the existing literature primarily has focused on parental psychological control and adolescent development. Psychological control undermines children’s autonomous function, thereby increasing the risk of social and psychological dysfunction (Barber and Harmon, 2002; Soenens et al., 2012). Adolescence is characterized by rapid separation and independence. It seems reasonable to assume the psychological control, which would hinder children’s independent development, exert the most distinct effects on adolescents. However, self-determination theory holds the psychological need for autonomy, competence, and relatedness present throughout life (Ryan et al., 2006). The negative impacts of parental psychological control would appear at various ages. Consequently, it would appear crucial to extend the body of existing research to include earlier developmental periods (Soenens and Vansteenkiste, 2010).

### The Joint Effects of Maternal and Paternal Psychological Control

Although the studies mentioned above included both parents’ psychological control, not all of them examined the joint effect or configuration (high or low levels of psychological control of both parents, or mixed levels of paternal and maternal psychological control) on children’s development. Therefore, the question remains of how children’s development may be impacted by discordant controlling parenting from their mothers and fathers.

With regard to the joint influences of parental psychological control, two distinct problems may be identified. The first is whether the existence of two highly psychologically controlling parents confers “dual risk” for children? The second is how the discordant parental psychological control (one parent employed high psychological control and another conducted low level of psychological control) influences the children development.

Although no research has been conducted to examine the combined effects of discordant parental psychological control on children development, several studies have examined concordant/discordant parenting patterns in relation to various constructs. For example, Simons and Conger (2007) claimed that children yielded the best outcomes in mental health and academic domains if both parents engaged in authoritative parenting and the worst outcomes if both parents engaged in non-authoritative parenting. Children with discordant parenting behaviors scored in the middle. Similarly, Kochanska and Kim (2013) examined the relationship between children’s attachment with their parents and future behavioral problems. The results showed that the children who had insecure attachment with both parents had significantly more behavioral problems than those who had secure attachment with both parents and those with mixed attachments. Having secured attachment with at least one parent appeared to have a significant beneficial effect.

### The Aims of This Study

In addition, the present study has two aims. First was to explore the relative predictive value of maternal and paternal psychological control on the socioemotional development of preschool children aged 2 to 5 in contemporary Chinese families, using the two indicators of behavioral problems and prosocial behaviors. Second was to investigate the joint effects of maternal and paternal psychological control on children’s socioemotional development.

## Materials and Methods

### Participants

Study subjects were recruited from four kindergartens in Beijing, the capital city of China. A total of 325 preschool children (169 girls and 156 boys) and their parents participated in the study. The selection criteria were as follows: (a) the parents were married and (b) the household was comprised of a nuclear family. One parent from each family provided written agreement to participate the research.

### Measures

#### Demographic Characteristics

Child’s age and gender, parents’ education, monthly family income, and family subjective social status (SSS) were collected. A total of 313 of these parents provided their educational levels: 24.3% of the children’s mothers had a Master’s degree or higher = 3, 63.1% had a bachelor’s or vocational college degree = 2, 10% had secondary education or less = 1 (*M* = 2.13, *SD* = 0.58), compared with 26.8, 63.95, and 9.3%, respectively, for fathers (*M* = 2.18, *SD* = 0.58). The *t*-tests were conducted to compare the families with full data and the families with missing data in terms of paternal and maternal psychological control and children’s socioemotional development. There were no differences between the two forms of families (in terms of paternal psychological control, *M*_full data_ = 2.20, *M*_missing data_ = 2.40, Δ*M* = 0.19, 95% CI = [-0.17, 0.56], *t* = 1.03, *ns*; in terms of maternal psychological control, *M*_full data_ = 2.12, *M*_missing data_ = 2.40, Δ*M* = 0.27, 95% CI = [-0.07, 0.61], *t* = 1.54, *ns*; in terms of children’s behavioral problems, *M*_full data_ = 0.52, *M*_missing data_ = 0.55, Δ*M* = 0.03, 95% CI = [-0.10, 0.17], *t* = 0.47, *ns*; and in terms of children’s prosocial behaviors, *M*_full data_ = 1.47, *M*_missing data_ = 1.43, Δ*M* = -0.04, 95% CI = [-0.26, 0.19], *t* = -0.33, *ns*).

A total of 308 of these parents provided their family monthly income: 6.8% of the families had monthly income no more than 6000 Yuan, 11.0% had monthly income from 6000 to 10000 Yuan, 31.5% had income from 10000 to 20000 Yuan, and 50.5% had monthly income more than 20000 Yuan. The *t*-tests were conducted to compare the families with full data and the families with missing data in terms of paternal and maternal psychological control and children’s socioemotional development. There were no differences between the two forms of families (in terms of paternal psychological control, *M*_full data_ = 2.22, *M*_missing data_ = 2.04, Δ*M* = -0.17, 95% CI = [-0.45, 0.10], *t* = -1.11, *ns*; in terms of maternal psychological control, *M*_full data_ = 2.14, *M*_missing data_ = 1.97, Δ*M* = -0.18, 95% CI = [-0.47, 0.11], *t* = 1.12, *ns*; in terms of children’s behavioral problems, *M*_full data_ = 0.52, *M*_missing data_ = 0.51, Δ*M* = -0.01, 95% CI = [-0.13, 0.10], *t* = -0.25, *ns*; and in terms of children’s prosocial behaviors, *M*_full data_ = 1.47, *M*_missing data_ = 1.53, Δ*M* = 0.06, 95% CI = [-0.13, 0.25], *t* = 0.66, *ns*).

Subjective social status (SSS) was defined as the individual’s perception of his own position in the social hierarchy (Sakurai et al., 2010). Several studies suggested that SSS may represent a stronger predictor of psychological stress (Sakurai et al., 2010) and children’s self-rated health (Goodman et al., 2007). The MacArthur Scale was adopted to assess parents’ SSS. A drawing of a ladder with 10 rungs was shown to participants with the description as follows: “the ladder represents the social classes where people stand in our society. At the top of the ladder are the people who are the best off, those who have the most income, most education, and best jobs. At the bottom are the people who are the worst off, those who have the lowest income, least education, and worst jobs or no job.” The participants were asked to mark the rung that best represents where they believe they stand on the ladder (Adler et al., 2000). As few parents choose the top and bottom rank, the 1∼3 ranks combined into low SSS group (coded as 1), 4∼7 ranks into middle SSS group (coded as 2), and 8∼10 SSS into high SSS group (coded as 3). Among the 303 parents provided this information, 4% parents chose low SSS, 87.5% chose middle SSS, and 8.6% chose high SSS. The *t*-tests were conducted to compare the families with full data and the families with missing data in terms of paternal and maternal psychological control and children’s socioemotional development. There were no differences between the two forms of families (in terms of paternal psychological control, *M*_full data_ = 2.21, *M*_missing data_ = 2.20, Δ*M* = 0.00, 95% CI = [-0.27, 0.28], *t* = 0.03, *ns*; in terms of maternal psychological control, *M*_full data_ = 2.14, *M*_missing data_ = 2.08, Δ*M* = 0.06, 95% CI = [-0.20, 0.31], *t* = 0.43, *ns*; in terms of children’s behavioral problems, *M*_full data_ = 0.52, *M*_missing data_ = 0.50, Δ*M* = 0.02, 95% CI = [-0.09, 0.12], *t* = -0.04, *ns*; and in terms of children’s prosocial behaviors, *M*_full data_ = 1.47, *M*_missing data_ = 1.47, Δ*M* = 0.00, 95% CI = [-0.17, 0.17], *t* = 0.30, *ns*).

#### Psychological Control

Psychological control was assessed with the Chinese version of the 18-item measure developed by Wang et al. (2007): 10 items identify guilt induction (e.g., *I tell my child that he/she should feel guilty when he/she does not meet my expectations*), 5 identify the withholding of love (e.g., *I act cold and unfriendly if my child does something I do not like*), and 3 identify authority assertion (e.g., *I tell my child that what I want him/her to do what best for them and not question authority*). Both fathers and mothers indicated how true each item was for themselves (1 = not at all true; 5 = very true). The psychological control value is the mean score derived from the 18 items: higher scores indicate greater psychological control. In this study, Cronbach’s alphas for mothers and fathers were 0.88 and 0.87, respectively.

#### Socioemotional Development

The 25-item Chinese version of Strengths and Difficulties Questionnaire (SDQ) was used to assess behavioral problems and prosocial behaviors as two indicators that reflected children’s socioemotional development levels. This scale was created by Goodman (1997), and Du et al. (2006) revised the original to create a valid and reliable Chinese version. The instrument may be used to assess behavioral and emotional problems of children and adolescents. The SDQ includes five subscales: emotional symptoms subscale, conduct problems subscale, hyperactivity subscale, peer relationship subscale, and prosocial behavior subscale. The scores for the first 4 subscales can be summed to generate a total difficulties score (Goodman, 1997; Silva et al., 2015) and we took the mean of the first 4 subscales as an indicator of behavioral problems. Mothers were asked to rate their children on a 3-point scale (0 = does not apply, 1 = partially applies, and 2 = certainly applies). In the current study, Cronbach’s alphas for behavioral problems and prosocial behaviors scales were 0.74 and 0.68, respectively.

## Results

### Preliminary Analysis

We examined the correlations among the measured constructs and represented them in **Table 1**. Maternal psychological control was positively related to behavioral problems and negatively related to prosocial behaviors. Paternal psychological control had a significant positive correlation with children’s behavioral problems, whereas correlation with prosocial behaviors was not significant.

**Table 1:** Descriptive statistics and correlations for measures constructs.

Variables	*M*	*SD*	Range	1	2	3
Behavioral problem^a^	0.52	0.23	[0.05, 1.20]	1		
Prosocial behavior^a^	1.47	0.38	[0.00, 2.00]	-0.34^∗∗^	1	
Maternal psychological control^a^	2.13	0.59	[1.00, 3.67]	0.31^∗∗^	-0.14^∗^	1
Paternal psychological control^a^	2.21	0.63	[1.00, 3.72]	0.18^∗∗^	-0.08	0.38^∗∗^


*^a^*n* = 325; ^∗^*p* < 0.05, ^∗∗^*p* < 0.01.*

To examine the associations between the measured structures and demographic characteristics, we conducted analyses of variance (ANOVAs) with maternal and paternal psychological control, children’s behavioral problems and prosocial behaviors as the respective dependent variables and child gender, maternal and paternal education, and SSS as the between-subject factors. In terms of prosocial behaviors, the main effect of child gender was significant, with girls exhibiting more prosocial behaviors than boys (*M*_boy_ = 1.41, *SD* = 0.38, *M*_girl_ = 1.52, *SD* = 0.38, Δ*M* = -0.11, 95% CI = [-0.20, -0.03], *F*(1,323) = 7.26, *p* < 0.01). With regard to behavioral problems, the main effect of SSS was significant [*F*(2,300) = 4.28, *p* < 0.05]. The estimated marginal means and 95% CIs for behavioral problems were 0.63 [0.50, 0.77] for the children with low SSS, 0.53 [0.50, 0.55] for the children with middle SSS, and 0.41 [0.32, 0.50] for children with high SSS. The children with high SSS displayed less behavioral problems than children with middle SSS (difference of estimated marginal means was -0.11, *SE* = 0.05, 95% CI [-0.21, -0.02], *p* < 0.05) and less than those with high SSS (difference of estimated marginal means was -0.22, *SE* = 0.08, 95% CI [-0.38, -0.06], *p* < 0.05). Therefore, the children’s gender and SSS were controlled in subsequent analyses.

### Concordance Analysis between Maternal and Paternal Psychological Control

To examine the difference between maternal and paternal psychological control, a paired-samples *t-*test was conducted. The results showed that paternal psychological control was significantly higher than maternal psychological control (*M*_m_ = 2.13, *SD* = 0.59, *M*_p_ = 2.21, *SD* = 0.63; Δ*M* = -0.07, 95% CI = [-0.15, 0], *t*(324) = 1.97, *p* = 0.05). K-means cluster was conducted to separate maternal and paternal psychological control into a high-score and low-score group and create four joint psychological control classifications: low scores of both maternal and paternal psychological controls (*n* = 124, 38.2%); high scores of both maternal and paternal psychological controls (*n* = 81, 24.9%); high scores of maternal psychological control and low scores of paternal psychological control (*n* = 50, 15.4%); and low scores maternal psychological control and high scores of paternal psychological control (*n* = 70, 21.5%), see **Table 2**. An examination of the concordance of maternal and paternal psychological control revealed considerable discordance (Cohen’s *k* = 0.25); 36.9% (120/325) of the children experienced discordant parenting psychological control context. Additionally, an examination of the correlation between maternal and paternal psychological control revealed a relatively positive relation, *r* = 0.38, *p* < 0.01.

**Table 2:** Classification of maternal and paternal psychological control.

	Paternal psychological control		
	
Maternal psychological control	High-score group	Low-score group	Total
**High-score group**	81	50	131
**Low-score group**	70	124	194
**Total**	151	174	325


### Socioemotional Development of Children in Different Groups

Descriptive statistics for children’s socioemotional development is displayed in **Table 3**. To test the connection between maternal and paternal psychological control and socioemotional development, one-way ANOVA was computed with the two indicators of socioemotional development as dependent variables and the four-way classification of psychological control as the between-subject factor.

**Table 3:** Descriptive statistics for children’s socioemotional development.

Outcomes	Classification (*n* = 325)										*F*(3,321)
		
	Total		L_F_-L_M_		L_F_-H_M_		H_F_-L_M_		H_F_-H_M_		
			(*n* = 124)		(*n* = 50)		(*n* = 30)		(*n* = 81)		
						
	*M*	*SD*	*M*	*SD*	*M*	*SD*	*M*	*SD*	*M*	*SD*	
Behavioral problems	0.52	0.23	0.44	0.23	0.58	0.23	0.53	0.21	0.60	0.23	9.90^∗∗∗^
Prosocial behavior	1.47	0.38	1.51	0.42	1.46	0.35	1.50	0.33	1.39	0.39	1.62


*L_*F*_-L_*M*_ = low paternal psychological control – low maternal psychological control; L_*F*_-H_*M*_ = low paternal psychological control – high maternal psychological control; H_*F*_–L_*M*_ = high paternal psychological control – low maternal psychological control; H_*F*_–H_*M*_ = high paternal psychological control – high maternal psychological control; ^∗∗∗^*p*<0.001.*

The data collected showed some interesting findings. The main effects of the organization of parental psychological control were significant for children behavioral problems [*F*(3,321) = 9.90, η^2^ = 0.09, *p* < 0.001]. The estimated marginal means and 95% CIs for behavioral problems were 0.44 [0.40, 0.48] for the children with both less controlling parents, 0.58 [0.52, 0.64] for the children with highly controlling mothers and less controlling fathers, 0.53 [0.47, 0.58] for the children with less controlling mothers and highly controlling fathers, and 0.60 [0.55, 0.65] for children with both highly controlling parents. Least significant difference (LSD) *post hoc* tests were used to examine pairwise differences among the four groups. Children in low maternal and paternal psychological control group had less behavioral problems than children in high maternal and paternal psychological control group (difference of estimated marginal means was -0.16, *SE* = 0.03, 95% CI [-0.22, -0.07], *p* < 0.01), and less than those who in low paternal psychological control – high maternal psychological control group (difference of estimated marginal means was -0.14, *SE* = 0.04, 95% CI [-0.22, -0.07], *p* < 0.01), and less than those who in high paternal psychological control – low maternal psychological control group (difference of estimated marginal means was -0.09, *SE* = 0.03, 95% CI [-0.15, -0.02], *p* < 0.01). Notably, however, there were no differences among the other three groups. Overall, children would be at risk of behavioral problems if they had one parent who deployed a high level of psychological control.

The estimated marginal means and 95% CIs for prosocial behaviors were 1.51 [1.44, 1.57] for the children with both less controlling parents, 1.46 [1.35, 1.57] for the children with highly controlling mothers and less controlling fathers, 1.49 [1.40, 1.59] for the children with less controlling mothers and highly controlling fathers, and 1.39 [1.31, 1.48] for children with both highly controlling parents. Psychological control classifications did not significantly impact prosocial behaviors [*F*(3,321) = 1.62, η^2^ = 0.02, *p* > 0.05]. There were no differences among the four groups with regard to prosocial behaviors.

### Regression Analysis for Psychological Control and Socioemotional Development

To examine the relative prediction and combined effects of maternal and paternal psychological control, hierarchical multiple regressions for each of the children’s socioemotional outcomes were conducted. The data were standardized before regression analysis and the simple size was 325. First, the child’s gender (0 = boy, 1 = girl) and SSS (the missing data were replaced by means) was entered, followed by maternal and paternal psychological control scores, and finally, the interaction terms of maternal and paternal psychological control in order to examine the combined effects of parental psychological control. The results were reported in **Table 4**. As expected, maternal psychological control could predict children’s behavioral problems positively (*b* = 0.06, 95% CI [0.04, 0.09], *p* < 0.05) and prosocial behaviors negatively (*b* = -0.04, 95% CI [-0.09, 0.00], *p* < 0.1), whereas paternal psychological control was not a significant predictor for the two indicators of children’s socioemotional development. Moreover, the interaction term significantly predicted children’s behavioral problems (*b* = -0.03, 95% CI [-0.05, -0.00], *p* < 0.05), but not their prosocial behaviors (*b* = 0.02, 95% CI [-0.03, 0.06], *p* > 0.1); these results were consistent with the ANOVAs.

**Table 4:** Regression analysis for parental psychological control predicting children’s socioemotional outcomes.

Predictors	Behavioral problems^a^					Prosocial behaviors^a^				
		
	*b* [95% CI]	*SE*	β	*R^2^*	Δ*R^2^*	*b* [95% CI]	*SE*	β	*R^2^*	Δ*R^2^*
**Step 1**										
Gender	-0.03 [-0.09, 0.02]	0.03	-0.07	0.04	0.04^∗∗^	0.12 [0.03, 0.20]	0.04	0.15*	0.02	0.02^∗^
SSS	-0.03 [-0.05, -0.02]	0.01	-0.19*			0.01 [-0.02, 0.04]	0.02	0.03		
**Step 2**										
MPC	0.06 [0.04, 0.09]	0.01	0.27*	0.13	0.09^∗∗∗^	-0.04 [-0.09, 0.00]	0.02	-0.11+	0.04	0.02^+^
FPC	0.01 [-0.01, 0.04]	0.01	0.05			-0.01 [-0.06, 0.03]	0.02	-0.04		
**Step 3**										
MPC × FPC	-0.03 [-0.05, -0.00]	0.01	-0.11*	0.14	0.01^∗^	0.02 [-0.03, 0.06]	0.02	0.49	0.04	0.00


*MPC, Maternal psychological control; FPC, paternal psychological control; SSS, subjective social status. The reported values are from the first time each variable entered the equation. ^a^*n* = 325; ^∗^*p* < 0.05, ^∗∗^*p* < 0.01, ^∗∗∗^*p* < 0.001, ^+^marginally significant.*

**FIGURE 1:**
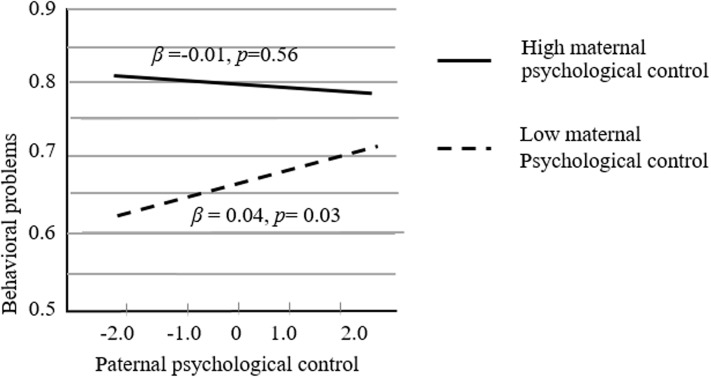
The joint effect of paternal and maternal psychological control (paternal psychological control as the independent variable and maternal psychological control as the moderator).

**FIGURE 2:**
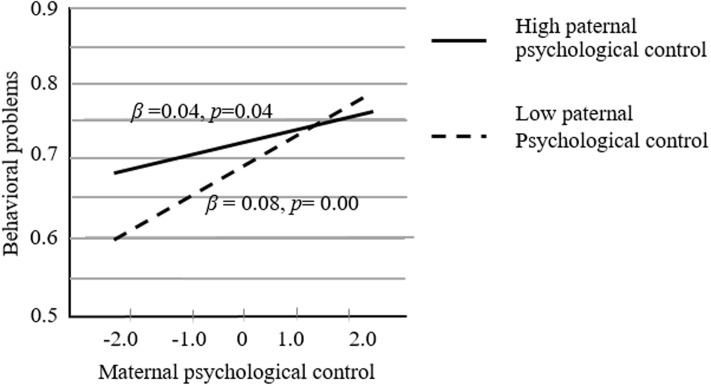
The joint effect of paternal and maternal psychological control (maternal psychological control as the independent variable and maternal psychological control as the moderator).

Follow-up simple slopes analysis was explored (Aiken and West, 1991). The graphical presentation of the product of maternal psychological control and paternal psychological control interaction is shown in **Figure 1** (paternal psychological control as the independent variable and maternal psychological control as the moderator) and **Figure 2** (maternal psychological control as the independent variable and maternal psychological control as the moderator). In **Figure 1**, the slope of the line representing high levels of maternal psychological control (+1 *SD* above the mean) was not significantly different from 0 [*t*(322) = -0.56, *p* = 0.56]. However, the slope of the line representing low levels of maternal psychological (-1 *SD* below the mean) was significantly different from 0 [*t*(322) = 2.13, *p* < 0.05]. For the children with a less psychologically controlling mother, high paternal psychological control was associated with an increase in behavioral problems. Nevertheless, the association was not significant for the children whose mother employed high levels of psychological control. In **Figure 2**, the slopes of the lines representing high levels of paternal psychological control (+1 *SD* above the mean) and low levels of paternal psychological control (-1 *SD* below the mean) were both significantly different from 0 (*b*_high_ = 0.04, *SE* = 0.02, *t*(322) = 2.09, *p* < 0.05, and *b*_low_ = 0.09, *SE* = 0.02, *t*(322) = 4.96, *p* < 0.001). Despite the level of paternal psychological control, maternal psychological control would consistently and positively predict children’s behavioral problems.

## Discussion

This study examined the predictive power and combined effects of maternal and paternal psychological control on the socioemotional development of preschool children 2–5 years of age in China.

Despite some researchers considered psychological control as a commonly used or acceptable parenting and thought it could promote children’s moral socialization (Helwig et al., 2014), our results found that psychologically controlling parenting was harmful to children’s socioemotional development in China. This result suggested that the detrimental effects of psychological control were culture-general. Therefore, parents even in China should reduce the use of parenting practices involving psychological control.

The most striking findings were the differential effects of paternal and maternal psychological control. Results revealed that higher maternal psychological control could predict more behavioral problems significantly and less prosocial behaviors marginally significantly. There were no parallel findings for paternal psychological control. These results could imply that the effects of maternal psychological control on children’s socioemotional competence were more clear and robust than the effects of paternal psychological control in China. These findings were not consistent with previous research in other countries. For example, a cross-cultural study, which examined the association between parental psychological control and depressive symptoms in Belgian and South Korean adolescents, showed that both maternal and paternal psychological controls were significant indicators for childhood depression (Soenens et al., 2012). Additionally, Arim and Shapka (2008) found that both maternal and paternal psychological controls significantly predicted externalized behavioral problems of British, Colombian, and Canadian children.

Several explanations for the lesser effect of paternal psychological control could be offered. First, the involvement of Chinese fathers in early childhood was still low. Most fathers were engaged in work and spend less time with family and children. A study conducted in China reported that 78.5% fathers spend less than 2 h with their children every day. In 67.8% of families, mothers or grandparents were the main caregivers for preschool children (Wei, 2013, Unpublished). Therefore, the paternal controlling parenting may exert relatively weaker effects on preschool children’s socioemotional development. Second, several studies suggested that maternal and paternal parenting might have differential influence on various aspects of a child’s development (Laible and Carlo, 2004; Boldt et al., 2014). The current study only examined behavioral problems and prosocial behaviors as outcomes, while the paternal controlling parenting may be a significant predictor for other areas of children’s development. Father’s involvement may be important to the development of individual achievement, self-definition, and independence (Soenens et al., 2010). Research has found that, compared with maternal parenting, paternal behaviors have a greater influence on a child’s self-esteem and self-efficacy (Bean et al., 2003). Therefore, paternal controlling parenting might play a greater role for children’s development in the areas beyond those examined in this study. Third, the relationship between paternal psychological control and children’s socioemotional development may be moderated by other variables. For example, Murray et al. (2014) suggested that the quality of the mother–child relationship moderated the association between paternal psychological control and children’s aggressive behaviors. More specially, paternal psychological control predicted children’s aggression when they experienced low-quality relationships with their mothers. Consequently, the main effect of paternal psychological control on children’s socioemotional development could be hidden in interactions between controlling parenting and other factors.

The results of this study suggested that there was no difference among the four groups with regard to prosocial behaviors. However, children whose parents employed less psychological control showed less behavioral problems than children in the other three groups. The findings indicated that children would be at risk for behavioral problems as long as one parent engaged in high levels of psychological control. Moreover, the interaction of paternal and maternal psychological control was significant for the prediction of children’s behavioral problems. The graphical representation of the interaction clearly suggested that a combination of less psychological control by both parents resulted in the fewest behavioral problems. Interestingly, the effect with maternal control as the primary agent and paternal control as the moderator suggested that maternal psychological control was consistently and positively associated with children’s behavioral behaviors no matter the character of paternal psychological control. In contradistinction, paternal control as the main agent and maternal control as the moderator showed that an increase of paternal psychological control was associated with an increase of behavioral problems when maternal psychological control was low. However, that was not the case when maternal psychological control was high. These results contribute to an understanding of the specific mechanisms at work with paternal and maternal psychological control. Maternal psychological control exerts a consistently negative effect on children’s development. Paternal psychological control matters only in conjunction with less maternal psychological control.

Although the current study provided a better understanding of the different effects of paternal and maternal psychological control, several limitations of this study should be noted. First, the cross-sectional nature of the current study prevents us to clear the direction of effects between parental controlling parenting and children’s developmental problems. Longitudinal research would be needed to test the causal relationships among these variables. Second, the sample was relatively homogenous. All of them were recruited from Beijing, the political, economic, and cultural center of China. Almost half of the families jointed the study were at high socioeconomic status. Parents showed relatively low level of psychological control and children were well-behaved. Therefore, the low level of variability of variables reduced the ecological validity of this study. Future studies could make a complete examination of parental psychological control and children development in heterogeneous population. Third, the data on parental psychological control and children’s socioemotional development were obtained from parental self-reports; the relationships identified could reflect common method variance. Therefore, future studies could utilize different measuring tools and informants to assure greater validity of the results. Fourth, the possible interplay between parenting and children’s characteristics could exist. The differential susceptibility hypothesis assumes that some children have high developmental plasticity and will be more affected by parenting experiences than others due to their own characteristics (e.g., temperamental, physiological, or genetics; Belsky and Pluess, 2013; Belsky et al., 2015). Numerous researchers have noted that children’s temperaments had a moderate effect on the association between parenting and development. For example, Bradley and Corwyn (2008) suggested that there would be a stronger relationship between maternal sensitivity and children’s behavioral problems among children with difficult temperaments. Zarra-Nezhad et al. (2014) found that children who displayed a relatively high level of social withdrawal were more vulnerable to the negative effects of high parental psychological control. Therefore, future research should consider the moderating role of children’s characteristics in the relationship between controlling parenting and children’s development. Finally, this study primarily focused on the parental psychological control, a family predictor of children development. However, teachers are alternative caregivers and teacher–child relationship is another important factor in preschool children’s socioemotional development (Burchinal et al., 2002; Murray et al., 2008). Future study would take the family and school factors into consideration and make a more comprehensive exploration about children development.

Despite these limitations, several strengths of the study merit mention. First, the research benefited from data collected on psychological control by both mothers and fathers from the same families. This provided the opportunity to examine the relative effect and joint effects of paternal and maternal psychological control on children’s socioemotional development. Second, the findings expanded upon existing knowledge regarding paternal psychological control and children’s development, and contributed a greater understanding of the practical implications. Parental psychological control had adverse effects on preschool children’s socioemotional development in Chinese culture. The children were at the high risk of behavioral problems as long as one parent exerts high level of psychological control. Therefore, we recommend that both parents, especially mothers, should reduce their controlling parenting and employ more adaptive parenting to promote children’s socioemotional development.

## Ethics Statement

This study was carried out in accordance with the recommendations of the Research Ethics Committee of Capital Normal University with written informed consent from all subjects. All subjects gave written informed consent in accordance with the Declaration of Helsinki. The protocol was approved by the Research Ethics Committee of Capital Normal University.

## Author Contributions

SX and XL conceived and designed the study. XG, XS, DZ, MZ, and BD performed the collection of data. XG analyzed and interpreted the data. SX, XL, and XG completed and modified the manuscript. MA provided language editing for the manuscript.

## Conflict of Interest Statement

The authors declare that the research was conducted in the absence of any commercial or financial relationships that could be construed as a potential conflict of interest.
